# Co-design of a Mobile Stroke Unit pathway highlights uncertainties and trade-offs for viable system-wide implementation in the English and Welsh NHS

**DOI:** 10.1186/s12873-025-01243-7

**Published:** 2025-06-08

**Authors:** L. Moseley, P. McMeekin, M. Allen, G. A. Ford, M. James, A. Laws, S. McCarthy, G. McClelland, L. J. Park, K. Pearn, D. Phillips, C. Price, L. Shaw, P. White, D. Wilson, J. Scott

**Affiliations:** 1 Faculty of Health and Life Sciences, Northumbria University, Newcastle Upon Tyne, UK; 2 University of Exeter Medical School, Exeter, UK; 3 NIHR South West Peninsula Applied Research Collaboration (ARC), Plymouth, UK; 4 Oxford University Hospitals NHS Foundation Trust, Oxford, UK; 5 Radcliffe Department of Medicine, University of Oxford, Oxford, UK; 6 Royal Devon and Exeter Hospital, Exeter, UK; 7 East of England Ambulance Service NHS Trust, Cambridgeshire, UK; 8 Stroke Research Group, Population Health Sciences Institute / Translational and Clinical Research Institute, Newcastle University, Newcastle Upon Tyne, UK; 9 Stroke Service User Voice Group, Newcastle Upon Tyne, UK

**Keywords:** Mobile stroke unit, Pathway development, Stroke, Co-production, Nominal group technique, Emergency medicine, Pre-hospital care

## Abstract

**Background:**

Mobile stroke units (MSUs) are specialist ambulances equipped with scanning and point of care testing that can identify patients eligible for intravenous thrombolysis – medication to dissolve a clot used in ischaemic strokes – and provide this on location. While benefits of MSUs have been demonstrated, this is context dependent. Routine use of MSUs across the English and Welsh National Health Service (NHS) has not yet been considered, and as such no pathway for their operation exists. This study aimed to co-design a viable pathway, detailing dispatch, staffing and treatment decisions, for MSUs within the NHS context.

**Methods:**

The study used interdisciplinary co-design alongside Nominal Group Technique (NGT) to generate consensus. Participants were recruited using a combination of purposive, opportunistic and snowball sampling. Data collection took place in online workshops, across three rounds, with supplemental interviews conducted where required. Data were analysed as an ongoing process, with participants checking interpretations after each round, and then further analysed deductively to identify key uncertainties following all the rounds. Consensus threshold for the NGT was set a priori at ≥ 80%.

**Results:**

An MSU pathway that reached consensus for being viable within the NHS was developed with consideration for current systems and pressures. Key uncertainties were identified such as where to base the MSU. We also identified where participants had to make trade-offs in the co-designed pathway, such as staffing considerations. Together, the uncertainties and trade-offs represent challenges to MSU implementation and are presented alongside the process to reach the finalised pathway. Future developments which may have implications for the implementation of MSUs were also explored.

**Conclusions:**

The co-designed MSU pathway provides a foundation for MSU implementation in the English and Welsh NHS and can be subjected to local and regional modifications required for implementation. However, optimal implementation is likely hindered by several uncertainties and trade-offs, including the geographical base of the MSU and staffing, that represent challenges to implementation of MSUs at scale. Future developments in acute stroke care may help to mitigate these challenges, such as developments in artificial intelligence to read scans and improved access to telemedicine.

**Clinical trial number:**

Not applicable.

**Supplementary Information:**

The online version contains supplementary material available at 10.1186/s12873-025-01243-7.

## Introduction

In the United Kingdom (UK) there are over 100,000 strokes every year and is one of the leading causes of death and disability [1]. It is estimated that the number of strokes in the UK is likely to rise by 59% by 2035 [2]. Mobile Stroke Units (MSUs) are specialist vehicles, equipped with scanning capabilities and point of care testing, which respond to suspected strokes alongside, or in place of, emergency medical services (EMS) [3]. The exact configuration of MSUs differs between trials with variations in staffing and the use of telemedicine, allowing for video calling with stroke physicians and the digital transfer of scans. There has also been differences in scanning with some including a computed tomography (CT) scanner, and others including computed tomography angiography (CT-A), with the latter using contrast to produce images of blood vessels [4–11]. Current studies of MSUs have shown that they reduce the time for someone to receive intravenous thrombolysis (IVT) given in some cases for the treatment of ischemic stroke to dissolve a blood clot [6–10]. Further benefits have been shown to include – in a limited number of studies – quicker access to mechanical thrombectomy (MT) [4, 5, 11] which is appropriate for some patients where a clot is occluding a large vessel (LVO) in the brain. Current care requires most patients eligible for MT to be transferred from a local acute stroke unit to a regional centre (comprehensive stroke centre (CSC)) capable of providing MT treatment, which can lead to a delay in receiving MT. MT can only be provided at CSCs due to the nature of the surgical procedure, which is relatively new, requiring highly trained interventionists of which there is a shortage [12].

While studies have shown potential benefits of MSUs [4–11] it is clear that this is context dependent [13]. Studies of MSUs have largely taken place in urban centres where, arguably, the population would already have quick access to stroke care and thus may contribute to existing inequities in access to stroke care that exist between rural and urban areas [14]. In England and Wales there are 12 EMS (known as ambulance services) covering large geographic areas, often across both urban and rural settings. In the UK, National Health Service (NHS) care is impacted by both high-level central government decision making, as well as each of the devolved nations (Wales, Scotland and Northern Ireland), making their own service decisions. NHS England and the Welsh NHS have a cross-border working agreement [15] with patients routinely accessing services in either country, particularly in relation to stroke care where Welsh patients, who require a CSC, can be closer to a CSC in England. For this reason, the study focused on NHS in England and Wales but it was not possible to include Scotland and Northern Ireland due to differing infrastructures and policies. While there are some health systems that operate similarly to NHS, successful implementation and changes cannot be solely made on comparison of how a health system operates. Other key features such as social and economic conditions, which determine public health, key gaps in health service provision and funding also impact heavily [16]. While there is currently one MSU being trialled in England [18], this is part of a single-unit research trial and as such cannot be fully relied upon in consideration of how MSUs could be implemented within the NHS. However, qualitative work with stakeholders has identified acceptability of MSUs in England and Wales providing that key implementation challenges can be resolved [19]. Further, existing studies have also differed in their approach to the MSU with different scanning capabilities, geographical radius serviced, and staffing configuration [4–11].

Clinical pathways are structured care plans, linking evidence to practice, to define the treatment for patients with specific clinical presentations, and are routinely used within the NHS [20]. Pathway development is an important component of designing and implementing new services for patients with a specific condition or set of symptoms, helping to ensure that service delivery is evidence-based [21] and increasingly person-centred [22]. Standardising care pathways also has the ability to reduce variations in care [23], which in turn can reduce inequalities in access [24]. Good pathway development requires a joined-up approach in its development. The development and successful implementation of a new care pathway can be a complex process with numerous barriers and facilitators across all stages of the process, including design [25]. While multiple MSU pathways have already been developed in other countries these are not directly transferable to other health systems, including the NHS as previously described. Context is recognised as vital for not just successful development and implementation of innovations, but to avoid harms that may occur if context is not considered [26]. Guidance from the Medical Research Council recognises the importance of potential new interventions not just being developed in collaboration but that they are also grounded in the context they would be implemented [27] One of the ways this can be accomplished is through the use of co-design. Co-design brings together a group of stakeholders, with expertise in a particular area, to discuss and ultimately influence how services are designed, implemented or in some cases commissioned [28]. One of the key elements of co-design is the involvement of patient and public involvement (PPI) representatives with lived experienced [29]. PPI has been underutilised in stroke research despite being an important element of service delivery [30] and a key consideration for the NHS [31]. As such the aim of this research is to co-design an MSU pathway, with key stakeholders including PPI representatives, in the English and Welsh NHS using a consensus-based approach, and to identify where uncertainties exist and trade-offs are made during co-design of the developed MSU pathway.

## Methods

### Study design

The study developed an MSU pathway over a series of interdisciplinary co-design workshops, including the use of a modified Nominal Group Technique (NGT) approach to generate consensus. Whilst a standard NGT is conducted in a single period of data collection [32], this was modified to conduct the NGT across three workshops to allow for more in-depth discussion and refinement of the MSU pathway prior to examining consensus. Supplementary interviews were used where participants were unable to attend workshop(s) to ensure inclusivity.

### Participants and recruitment

Recruitment strategies utilised included purposive, opportunistic and snowball sampling of relevant stakeholder groups (stroke clinicians (physicians, nurses), ambulance staff, stroke patients or those supporting stroke patients and commissioners). Practitioners were identified through existing networks of the research team, who contacted known individuals who worked, researched or had other involvement with stroke care. Once identified an initial email was sent to the identified potential participants outlining the study details and contact the research team if they would like to participate. Potential participants were also invited to share the email with others who may be interested in taking part and the sample was recruited from all those who responded to the invitation. Patient and public participants were recruited via a charity, The Stroke Association, who support people who have experienced stroke and those impacted by stroke. To recruit patient and public participants, a gatekeeper sent a lay summary of the research to people who had expressed an interest in supporting research, consisting of stroke survivors, carers, and/or family members of people who had experienced stroke. Potential participants were asked to express an interest, from which they were purposively sampled based upon geographical location, age, gender and type of stroke to ensure it was a representative sample.

### Data collection

All data were collected online using Microsoft Teams with a total of three rounds of data collection. All discussions were audio recorded. Data collection took place between November 2023 and May 2024. PPI representatives were supported by individual meetings with one of the researchers (LM) prior to the first workshop to explain the research format, provide an overview of MSUs, and give an overview of the current NHS stroke pathway. The PPI representatives were given the opportunity to ask questions as well as highlight any ways we could ensure the workshops were accessible, including any individual accessibility requirements. Further, a document was produced which provided key terminology and acronyms, with an explanation which was kept updated throughout the workshops. Prior to data collection, a topic guide was developed for use in this study (Supplementary material 1) including prompts to cover key aspects of an MSU pathway that was based on the expertise of the research team. This topic guide was used to facilitate rather than guide discussion. Prompts included: dispatch protocols; staffing/crewing; role of telemedicine; nature of imaging; treatment eligibility criteria; protocols; treatment options for rural / urban patients and access to hospital services; infrastructure costs; alternative or interacting care pathways (e.g. stroke mimics); does anything discussed introduce or address potential inequalities in access and conveyance location. Both professional and PPI stakeholders attended the same workshop. Where data were collected via interview, all interviews in that round were held post-workshop, and the researchers gave viewpoints that arose in the workshop to act as discussion prompts. Data collection was led by JS with input from other members of the research team (LM, MA, PM, GM, LP).

#### Round 1

Participants received an initial presentation which included a description of existing MSUs with example videos of MSUs in England [33] and United States [34], followed by clarification questions from participants to ensure understanding of MSUs. The workshop then moved to open-ended discussion about MSUs, including questions about what should be considered when establishing an MSU. Data were additionally recorded using an interactive Miro whiteboard. Following the first round of data collection, the research team created two initial MSU pathways with a red, amber, green coded list of pathway elements to express (un)certainty by drawing on workshop data. Red coded items represented areas where there was a high level of uncertainty about a particular element of the pathway, with amber having some uncertainty and green being no uncertainty. This approach allowed us to identify areas where discussions should be focused.

#### Round 2

The two initial MSU pathways were presented to participants, with an opportunity to ask clarifying questions. Participants then discussed the different elements of the pathway, with specific emphasis on those parts of the pathway where there was greatest uncertainty. Participants were asked to individually identify and comment on parts of the pathway where they felt that revisions, omissions and/or clarifications were required (as part of the NGT approach), along with justifications in order to understand reasons for uncertainty and how they could be overcome. These were then used as prompts for additional discussion amongst all participants. Data were additionally recorded in tables, shown live to the participants for ongoing member checking. Pathway revisions were again made by the research team following the second round of data collection, with a single MSU pathway developed.

#### Round 3

A second iteration of the MSU pathway was presented, with an opportunity for participants to ask clarifying questions. Prior to any discussion, the different elements of the pathway were placed into an online survey, where participants were asked to rate on a 5-point Likert scale (strongly disagree to strongly agree). Participants were also asked where the MSU should be based with the option of the ambulance service or a comprehensive stroke centre, and how important this was on a 5-point Likert Scale (very unimportant to very important). Ratings were then compiled within the workshop and used to facilitate final discussion on justifications for answers; this included discussion of where consensus was achieved, albeit with a primary focus on where there was no consensus. Interviews conducted post-workshop used the same approach, with participant data adding to existing consensus scores. Discussion then focused specifically on either deviation from the existing data (i.e. participants agreeing with a pathway element where there was not previously consensus) or where consensus was not achieved.

### Data analysis

Analysis was an ongoing process throughout all rounds of data collection, with participants commenting and reflecting on any interpretations by the research team as part of mapping the pathway. This followed best practice in co-design, where participants were able to check the ongoing work and interpretations of the researchers, thus improving trustworthiness and rigour [35]. Following each round of data collection, audio recordings were transcribed verbatim and were analysed using Nvivo Version 12 (Lumivero) [36] by a researcher (LM) to chart the iterative development of the pathway.

For the NGT survey in round 3, the consensus threshold was set a priori as ≥ 80% of people either somewhat agreeing or strongly agreeing with items. One participant’s responses were excluded from certain questions within the survey as the participant advised they did not understand the particular element of the pathway and selected the ‘unsure’ option. Given the participant’s statements it was felt appropriate to exclude their data on those specific questions to ensure an accurate representation of consensus.

Upon completion of the final developed pathway, the research team (LM and JS) re-examined transcripts deductively to identify where participants had reported uncertainties and trade-offs relating to the final pathway elements.

## Results

### Participants

Fifteen people participated in round 1 and 2 and fourteen participated in round 3. One participant who contributed in round 1 was unable to continue with the study however was replaced by a participant in a similar role, in the same ambulance service, for rounds 2 and 3. One participant attended rounds 1 and 2 was unable to attend round 3 and was not replaced. All other participants remained the same across all the rounds. Participants were geographically dispersed, with good representation of areas across England, however there was only one participant from Wales. While the study aimed to recruit commissioners, this was unsuccessful. The research team were able to contact, and have informal discussions, with commissioners of various services (ambulance service; local NHS commissioners and national NHS commissioners) but none felt the commissioning of MSUs in the NHS, if they were to be implemented, would fit in their remit. Participant characteristics are shown in Table 1.

**Table 1:** Participant characteristics

Primary background	Round 1	Round 2	Round 3
*PPI Representative*,* n (%)*	6 (40)	6 (40)	6 (43)
*Stroke consultant*,* n (%)*	5 (34)	5 (34)	4 (28)
Ambulance service staff*,* n (%)*	4 (26)	4 (26)	4 (28)

* exact roles not reported to ensure anonymity, but consisted of people with both front-line clinical (nurses, paramedics) and senior management expertise

Round 1 workshop lasted 160 min with supplement interviews lasting between 28 min and 76 min (mean = 48). Round 2 workshop were completed in two sessions and lasted 135 min and 152 min (mean = 143). Round 2 supplemental interviews lasted between 39 min and 75 min (mean = 58). Round 3 workshop lasted 156 min with supplemental interviews lasting between 59 min and 88 min (mean = 78).

### Developed pathway

As a result of three rounds of pathway co-design, a single pathway was developed on which consensus was developed. The findings section of this manuscript reports on the final pathway, drawing on data collected from across the three rounds to evidence the various elements of the pathway. Data are reported together to highlight where uncertainties exist and trade-offs were made in the pathway development, as discussions often spanned multiple rounds.

The final workshop presented participants with the co-designed pathway with the only undecided factor being where the MSU would be based. In the pathway, the MSU would either have a static location at a CSC, or a dynamic location in the community. To generate consensus on the pathway elements, a survey presented 20 items, of which 14 (70%) items reached consensus. Six (30%) items did not achieve consensus, demonstrating remaining uncertainties in certain parts of the pathway. Specific pathway elements and levels of consensus are presented in Table 2. As consensus was defined as ≥ 80% the table condenses somewhat agree/strongly agree and somewhat disagree/strongly disagree. The following sections report on the discussions around uncertainties and trade-offs within the MSU pathway, then the final developed pathway is presented.

**Table 2:** Pathway elements consensus

Question				
	Comprehensive stroke centre	Ambulance Station		Consensus reached
*The MSU should be based at?*,* n (%)*	4 (29)	10 (71)		No
	**Very or somewhat unimportant**	**Neither important or unimportant**	**Very or somewhat important**	
*How important is it to you that the MSU is based here?*,* n (%)*	1 (7)	2 (14)	11 (79)	N/A
	**Strongly or Somewhat Disagree**	**Neither agree nor disagree**	**Strongly or Somewhat agree**	**Consensus reached**
***Staffing and equipment***				
*To what extent do you agree or disagree that it is suitable for the MSU to*:				
*Be staffed by a stroke nurse*,* radiographer*,* paramedic and ambulance technician?*,* n (%)*	0 (0)	2 (14)	12 (86)	Yes
*Be supported by a stroke physician via telemedicine?*,* n (%)*	1 (8)	0 (0)	13 (92)	Yes
*Have an ambulance technician onboard to drive the ambulance under blue light conditions allowing the paramedic to support with care in the mobile stroke unit?*,* n (%)*	2 (14)	0 (0)	12 (86)	Yes
*Be permanently staffed?*,* n (%)*	0 (0)	0 (0)	14 (100)	Yes
*Have a CT-A scanner?*,* n (%)*	0 (0)	0 (0)	14 (100)	Yes
*Operate at similar hours to the local thrombectomy service?*,* n (%)*	1 (7)	2 (14)	11 (79)	No
***Dispatch***				
*To what extent do you agree or disagree that it is suitable*:				
*For the MSU to be dispatched as an additional resource alongside a standard ambulance?*,* n (%)*	2 (14)	2 (14)	10 (72)	No
*That the team staffing the MSU would assess/listen to calls coming into to the ambulance service and decide whether to dispatch the Mobile Stroke Unit?*,* n (%)*	0 (0)	1 (7)	13 (93)	Yes
*That the additional triage by the MSU staff team would consider stroke calls as well as other symptoms that may indicate stroke*,* i.e. falls and severe sudden onset headaches?*,* n (%)*	0 (0)	2 (14)	12 (86)	Yes
*That a standard ambulance on scene would be able to request the MSU*,* where appropriate*,* if a stroke is suspected?*,* n (%)*	0 (0)	0 (0)	14 (100)	Yes
*That a standard ambulance and MSU could rendezvous enroute to the hospital if required?*,* n (%)*	3 (21)	0 (0)	11 (79)	No
*For the standard ambulance to begin triage on scene and the MSU ‘stood down’ if not required?*,* n (%)*	1 (7)	0 (0)	13 (93)	Yes
***On scene and conveyance***				
*To what extent do you agree or disagree that it is suitable*:				
*For the MSU to handover care to the standard ambulance on scene where no stroke is identified or where no specialist treatment is being provided by the MSU?*,* n (%)*	0 (0)	1 (7)	13 (93)	Yes
*For the standard ambulance to be ‘stood down’ when the MSU is providing care?*,* n(%)*	0 (0)	0 (0)	14 (100)	Yes
*For patients with a large vessel occlusion to be transported to the Comprehensive Stroke Centre?*,* n (%)*	0 (0)	1 (7)	13 (93)	Yes
*For patients who do not have a large vessel occlusion to be transported to the Acute Stroke Unit or the Comprehensive Stroke Centre*,* which ever is closest?*,* n (%)*	0 (0)	1 (7)	13 (93)	Yes
*That if there are any connectivity difficulties on scene impacting on telemedicine the patient would receive ‘standard care’ for a stroke?*,* n (%)*	5 (35)	0 (0)	9 (65)	No
***Overall***				
*To what extent do you agree or disagree*:				
*That pathway 1*,* with the MSU based at the comprehensive stroke centre*,* is viable in the NHS?*,* n (%)*	2 (15)	3 (23)	8 (62)	No
*That pathway 2*,* with the MSU based at the ambulance station*,* is viable in the NHS?*,* n (%)*	0 (0)	2 (15)	11 (85)	Yes

The pathway was explored in four parts: (1) location; (2) staffing and equipment; (3) dispatch; and (4) on-scene and conveyance. These four areas were developed over the course of the rounds based on how participants grouped discussions together.

#### Base location of MSU

Location did not receive consensus within the survey with four participants (29%) believing MSUs should be based from comprehensive stroke centres and ten participants (71%) believing a dynamic model based out of the ambulance service was more appropriate. When asked about the viability of the pathway within the NHS, wither based at a CSC or a dynamic model, majority of participants believed that the dynamic model based within the ambulance service was viable in the current NHS whereas the model based at the CSC was not viable.

The base location of the MSU was a key area of discussion during all three rounds. While previous studies have based them in urban areas, participants felt that this was not suitable or viable in the NHS. The main concern centred around CSCs not having the capabilities to manage the MSU, including dispatching as well as maintaining the vehicle. Further, the CSCs in England are based in dense urban areas, with participants reporting this would limit the areas the MSU would cover, including being able to provide a service to rural areas, and thus limit their ability to address geographic inequalities in access to stroke care. This is summed up by an ambulance service staff member:

*around a comprehensive stroke unit*,* they’re probably some of the best-performing areas already*,* so why?…why would you put something in there*,* in an area that’s already*,* probably*,* performing a lot better than an area where there is an ASU [acute stroke unit] only*,* or not any stroke services (Participant 4*,* ambulance service staff*,* workshop)*

However, it was also recognised that by placing MSUs in more rural areas the utilisation of the resource would be lower and not see as many patients. This conflict around location is highlighted by a PPI representative:

*My heart says stick it in the areas that are remote and difficult…[my] head says stick it in the major population centres where you’re going to get lots more cases for it to deal with. (Participant 17*,* PPI representative*,* workshop)*

#### Staffing and equipment

Staffing and equipment areas mainly reached consensus within the survey, with only MSU operating hours not achieving consensus. On discussion with participants they advised that thrombectomy services are moving toward 24 h services but did not feel the MSU would be of benefit through the night and would see a relatively small amount of patients, thus making it inefficient. This concern was explained by an ambulance service staff member:

*…there is a huge drive to get all thrombectomy centres 24/7 this year…I suppose*,* for me*,* it’s two things really*,* acknowledging the huge challenge there would be about staffing the mobile stroke unit 24/7…and appreciating the fact that nobody’s doing this [operating MSUs overnight] yet…We know that there is a lull in activity overnight… (Participant 5*,* ambulance service staff*,* workshop)*

While the staffing combination did reach consensus, this was the largest area of uncertainty and debate across all the workshops. It was agreed that having a stroke physician onboard would be ideal, but it was recognised that if MSUs were to be rolled out across England and Wales this position would unlikely be feasible. Participants explored numerous ways to include a stroke physician onboard, including having the MSU based at the CSC with a stroke physician on-call to staff the MSU as required, or having a regional rota of stroke physicians who would be dedicated to the MSU on their rota day. Staffing decisions significantly overlapped with the base location, detailed above, factoring into the discussions about whether MSUs should be based at CSCs to allow a stroke physician to join the vehicle. However, given the shortage of stroke physicians within the NHS it was felt this position would be untenable and therefore staffing considerations no longer impacted on base location decisions. The compromise of an experienced stroke nurse, alongside a paramedic, radiographer and another member of ambulance service staff could successfully staff an MSU with the support of a stroke physician via telemedicine. This is highlighted by a stoke consultant:

*…About 50% of posts are not filled with stroke consultants. To take a stroke consult away if they are on call…or if they are doing a ward round…if I’m a manager…I will not be happy. I’m going to challenge that…from a clinical perspective that is really going to be challenging*,* to have a consultant on that mobile stroke unit. My suggestion would be*,* instead of having a stroke consultant*,* can we have…a registrar or an ACP [advanced care practitioner] on the basis that*,* once the CT is done…images would be transported to the consultant who is in the building*,* like we do telemedicine? Then they can advise…whether to go ahead with thrombolysis or not. (Participant 13*,* stroke consultant*,* workshop)*

Two people did disagree with the staff configuration, however on discussion this was as a result of the ‘ambulance technician’ when this role could be fulfilled by lots of job roles within the ambulance service. This disagreement was therefore a result of wording choice and not concern for the overall configuration, and highlights that there would need to be some extent of service-specific configurations of MSUs that incorporate relevant terminology.

*…you could probably forego the technician and have a care assistant or a non-clinical driver in that role… (Participant 4*,* ambulance service staff*,* workshop)*

#### Dispatch

Dispatch of the MSU was another key area of uncertainty during the pathway development. Participants felt strongly that sensitivity and specificity of current ambulance triage needed to be improved for dispatching the MSUs to ensure attendance at the most appropriate calls, with participants assuming both that there would likely be very few MSUs and they would be a high-cost resource. Therefore, there was consensus that MSU staff could screen calls coming into the ambulance service and self-dispatch using a combination of the triage algorithm and their own clinical decision making. This could include calls for the most commonly triaged conditions that may indicate stroke but do not receive a stroke disposition, such as falls and severe sudden onset headaches.

While participants reached consensus on how the MSU would be dispatched, an area of disagreement was regarding dual dispatch. The workshops had lengthy discussions about the high probability that the MSU would attend at least some calls that were not stroke, regardless of what level of triage was in place. The participants discussed concerns that if the patient was not having a stroke that the MSU could spend a large amount of time treating a non-stroke patient and that due to this being a specialist resource this would not be an effective use. There was particular concern about them conveying non-stroke patients to hospital given the current waiting times ambulances are facing at the hospital. The dual dispatch model would send an ambulance as per standard care, and the MSU would be an additional resource that attends when it makes the decision to dispatch. While the majority of the participants were in agreement with this sentiment, non-consensus arose due to concerns that it would be difficult to justify two resources attending and whether this would be feasible given current pressures on ambulance services, as exemplified by an ambulance service staff member:

*…the model where you send both the conventional ambulance and the specialist unit is the European/American model*,* so there is quite a lot of literature around that. In most of those cases*,* the conventional ambulance is first on-scene. That could be doable in the NHS…but…they couldn’t really justify sending two ambulances to one job when we’ve got a hundred jobs that need an ambulance. (Participant 4*,* ambulance service staff*,* workshop)*

The other item that did not reach consensus in relation to dispatch was the idea of a standard ambulance rendezvousing with the MSU. A PPI representative and a stroke consultant elaborated on their concerns for this approach:

*I think it’s incredibly difficult to logistically organise rendezvous and build in delays…this is putting extra pressure on your dispatch team and the individual drivers*,* and it feels like rendezvous*,* as part of the approach*,* is going to raise a whole load of unintended challenges and consequences. (Participant 3*,* PPI representative*,* workshop)*

*I do not really agree. There’s no point having both of them going around*,* for me*,* from a resource implication point of view. (Participant 13*,* stroke consultant*,* interview)*

However, participants with experience of dispatching and working on ambulances explained that rendezvous already happens, for example with critical care doctors, and were therefore confident this could be implemented and managed.

#### On scene and conveyance

On scene and conveyance decisions were relatively unproblematic throughout the workshop with high levels of agreement on conveyance destinations dependent on the CT-A scan results, determining whether a patient has an LVO and should be transported to a CSC or whether there is no LVO identified and can be transported to an ASU. The only question that did not reach consensus was whether the MSU would revert to standard ambulance care where there were issues with telemedicine connectivity. This was added to the pathway to provide a contingency due to the inevitability of telecommunication being unavailable in certain parts of the country, particularly rural areas. Upon discussion of non-consensus items with participants it was advised that the disagreement was not due to the contingency being included, but that there was more local decision making needed rather than a blanket inclusion of reverting to standard care. For example, if the MSU strongly suspects a stroke, and from local geographic knowledge know that telecommunication would likely work if they moved a short distance, this would be the preferable option as highlighted by a stroke consultant:

*…If they can wait five minutes to get some connection*,* then they could do that. Or the other thing they could do*,* in terms of the connectivity*,* can they try other means of communicating with the stroke team? If it’s not online*,* can they use their phones or another form of communication? (Participant 13*,* stroke consultant*,* interview)*

One area that did generate a significant amount of discussion throughout the first and second rounds, which was resolved prior to the pathway being developed for round three and therefore not represented in the questions used for consensus, was in relation to haemorrhagic strokes. Haemorrhagic strokes are caused by a bleed in the brain rather than a clot seen in ischaemic strokes and therefore not suitable for treatment with IVT. While it was recognised that the MSU would be able to identify a haemorrhagic stroke, the treatment options available are limited, and that it is more important to get the patient to a hospital as soon as possible.

#### Developments that will inform future pathways

While not directly impacting on the developed pathway, another form of uncertainty expressed by participants was around the ongoing development of new technologies, medications and procedures that may inform future MSU pathways, but are not yet in routine practice. These remain worthy of consideration for future work. The current medication used for IVT in the NHS is alteplase which needs to be given as an infusion and requires a pump which cannot be used within a standard ambulance by paramedics. However, tenecteplase, which can be given as a bolus, is currently increasing in use for IVT which may mean the MSU would not need to stay with the patient once treatment started. This benefit was explored by an ambulance service staff member:

*I just wonder if that’s worth considering*,* at some point. Because if you can get to the point where you’re giving a tenecteplase bolus*,* you’re freeing up your mobile stroke unit. (Participant 5*,* ambulance service staff*,* interview)*

Current trials of medications commonly used for bleeding disorders being used for haemorrhagic stroke was a key area of development for one stroke consultant who felt that this could be something delivered on the MSU in future:

*The point about intracerebral haemorrhage is there are two large trials of basically pro-clotting*,* pro-coagulant drugs. (Participant 7*,* stroke consultant*,* workshop)*

Technological developments included connectivity improvements that are currently being trialled, and incorporation of artificial intelligence diagnostics that could affect staffing of the MSU, as well as more advanced scanning being developed. A stroke consultant discussed the AI potential and an ambulance service staff member shared their knowledge of current connectivity trials:

*The current generation…[of AI] are not great. But they are first generation*,* and most things in the first generation are not great. So I think*,* with better scanning quality and with later generations of AI*,* we’re going to be much better at making these diagnoses of particularly LVO (Participant 7*,* stroke consultant*,* workshop)*

*…[current connectivity trial] potentially overcomes that issue*,* because I think it has got the ability to access any of the mobile networks and if not*,* use satellite. (Participant 6*,* ambulance service staff member*,* interview)*

### Final MSU pathway

**Fig. 1:**
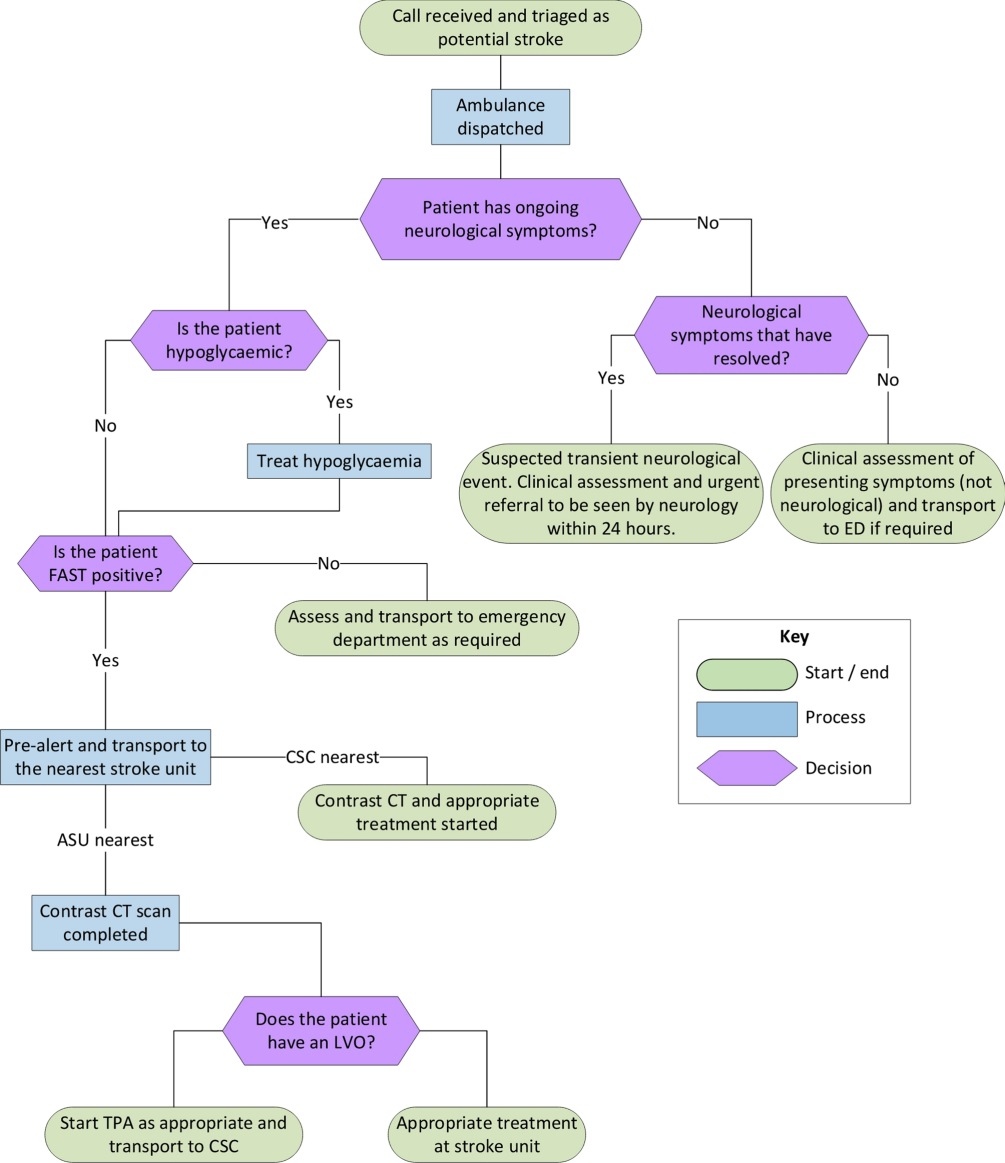
Existing pre-hospital acute stroke pathway

**Fig. 2:**
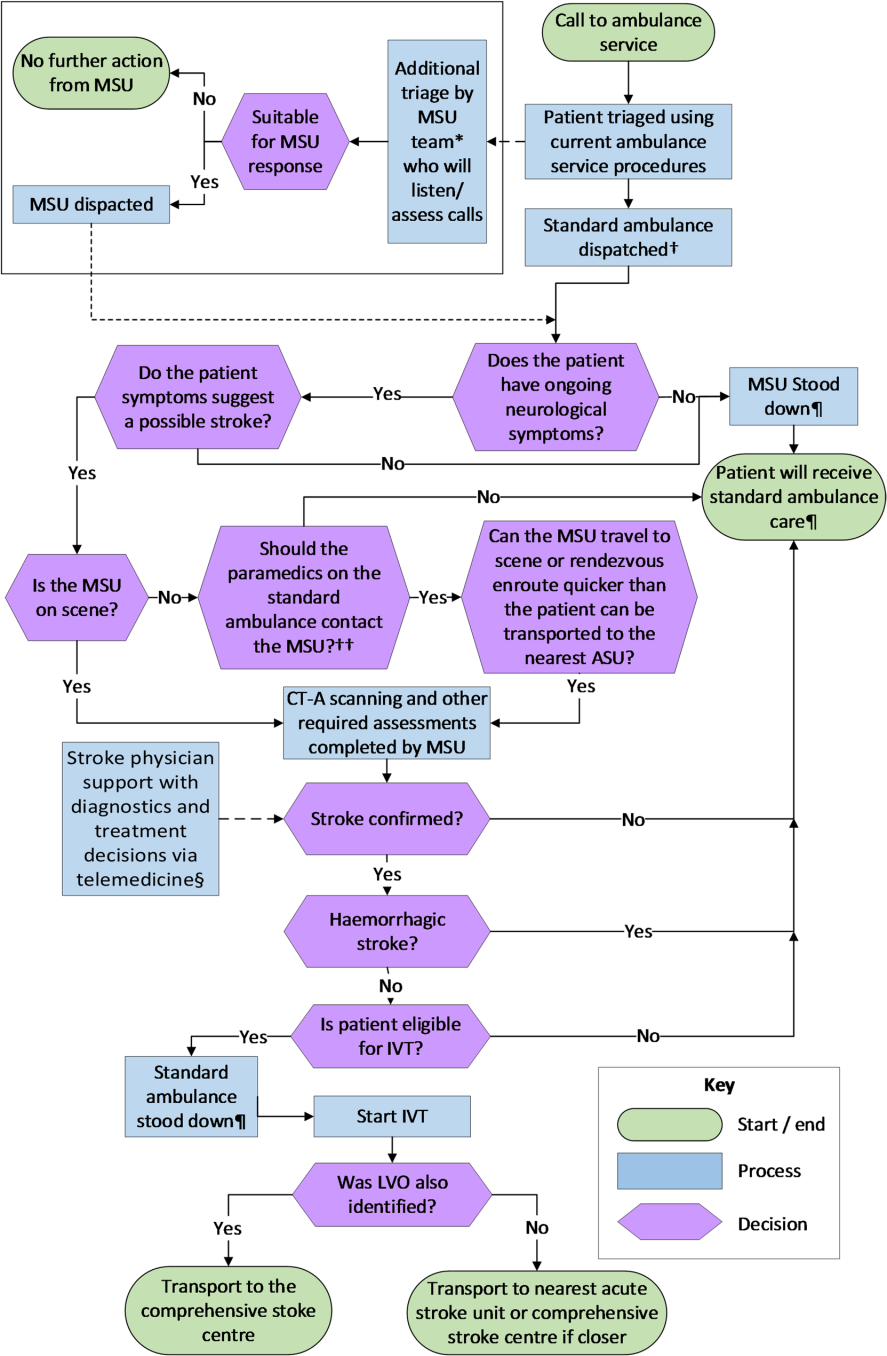
Finalised MSU pathway. * MSU would be staffed by an experienced stroke nurse, paramedic, radiographer and an additional member of ambulance service staff. This was a trade-off as participants preferred a stroke physician but recognised that the shortage of stroke physicians would not make this viable. † Dual dispatch has been included in the pathway however some participants raised concerns about the viability and justification of this given current resource constraints. Without dual dispatch it was recognised that the MSU could spend a significant amount of time treating non-stroke related patients. †† Local decision making would be required about the criteria for paramedics to contact the MSU. ¶ These elements are contingent on dual dispatch being implemented. Alternatively, if the MSU attended as a single resource to a none-stroke patient then local decision making would be required about whether a standard ambulance should be dispatched so the MSU could be ‘stood down’. § Telemedicine was a trade-off in relation to physician shortage concerns. Participants recognised that telemedicine would not always be reliable in certain geographical areas and local decision making would be required about whether there was an area within an acceptable distance that would have connection or whether they should revert to standard care

A summary of existing pre-hospital acute stroke pathways [37, 38] is presented in Fig. 1, and the final MSU pathway is presented in Fig. 2 with representation of uncertainty and trade-offs. The MSU would be owned and controlled by the ambulance service and could be optimally and dynamically positioned, based on local data, to best allow it to respond to suspected strokes. A dual dispatch system, with the MSU being dispatched alongside a standard ambulance, continued to be included in the final pathway. The benefit of the dual dispatch system is that the MSU could leave the scene where no stroke is identified, or they are unable to provide any specialist treatment. This would allow the MSU to attend more suspected strokes while leaving the patient with appropriate care. Alongside attending patients directly, the MSU would also be able to rendezvous with an ambulance in the community where this is indicated. The decision to rendezvous would, again, be based on local decision making but it is envisaged this option would be used, for example, if a standard ambulance responded to a suspected stroke in a rural area and could meet the MSU much quicker than going directly to hospital. The pathway developed details treatment options dependent on the patient presentation.

## Discussion

Whilst MSUs are being implemented in numerous countries, this is usually limited to single, urban locations, and MSU pathways are not always clearly articulated [4–11]. This is the first study to develop a standard MSU pathway applicable to the context of a whole health system (NHS), which was conducted using best-practice approaches to co-design and consensus development that adhere to the MRC framework for intervention development [39]. The result is a co-designed MSU pathway that is context-sensitive and adaptable for local and regional implementation, and which we contend has the greatest potential for viability in the NHS in England and Wales. The pathway can inform future research such as economic modelling of MSUs, clinical trials, or future implementation of MSUs, particularly via the identification of uncertainties and trade-offs identified during the pathway development.

The uncertainties and trade-offs highlighted in the results represent the complexity of the implementation MSUs but also offer, in some cases, acceptable alternatives. There was a strong debate regarding economic value versus inequality of care, with participants failing to reach a definitive conclusions, however more participants strongly expressed that MSUs in England and Wales should be located near those with the current worst access to care, likely rural or smaller urban areas, which is in line with previous research examining this dilemma [40]. The average population is older in rural areas in comparison to urban areas [41], and has high pockets of socioeconomic deprivation [42], both of which are significant risk factors for stroke [43, 44]. While it is recognised that socioeconomic deprivation is also present in urban areas, the combination of poor geographical access to health care, alongside the risk factors for stroke, make a compelling argument about placing MSUs in rural areas. However, the concern remained throughout that MSUs are unlikely to be funded if they could not also demonstrate an economic value to the NHS, achieved by seeing a larger number of patients [45] which would require an urban location, despite the NHS recognising equality of access to care as a priority [46]. The ownership and operation of the MSU by ambulance services allows for the location of the MSU to be dynamic and goes some way to addressing this difficulty, but requires additional demand modelling by individual ambulance services to determine locations that would provide optimal balance between equity of access and cost-effectiveness, two important components of care quality [47].

The pathway was developed with current NHS pressures at the forefront of thinking, introduced by the participants. The NHS has a shortage of stroke clinicians [48] which is only increasing [49] and while it would be ideal to have a stroke physician onboard the MSU it would likely make the pathway unviable. Similarly, current hospital pressures are increasing ambulance wait times outside hospitals to handover patients, which in turn is reducing the number of available ambulances [50]. This creates two concerns. Firstly, if the MSU is conveying all patients – regardless of having a stroke diagnosis or not – to hospital, there is a high likelihood that they would be unavailable to attend further suspected strokes if they are queuing outside the emergency department, particularly given the likelihood that the MSU would attend patients who, after assessment, did not have a stroke (e.g. mimics) [18]. The dual response model, with a standard ambulance being dispatched as standard and the MSU being an additional resource would provide some mitigation. This matches some helicopter emergency medical service (HEMS) dispatch, though there is a lack of high quality evidence on whether clinician-based dispatch improves dispatch decisions [51], and evidence suggests that medical dispatchers struggle to identify large vessel occlusions that would improve access to mechanical thrombectomy [52]. For MSUs, this did not reach consensus due to concerns of whether committing two resources is justified in the context of services that are already under pressure. However, this has continued to be included in the pathway as one resource can be ‘stood down’ quickly and this would be a more efficient use of the MSU where no stroke is identified. The uncertainty around this approach would require studying in either trials or as part of robust evaluation of MSU implementation should they be piloted or commissioned in the NHS in England and Wales.

Lastly, the workshops raised a number of future developments which may or may not influence the MSU pathway. If implementing an MSU pathway, people involved should determine whether these future developments have been actualised and to what extent they should inform the pathway. For instance, the expected increased use of tenecteplase in the coming year [53], which can be given as a bolus, would raise the question of whether the MSU would need to convey the patient. This could be managed within a standard ambulance within the current or updated skillset of paramedics, as has recently been demonstrated in a Norwegian setting where paramedics were deemed to provide sufficiently safe care following the provision of thrombolytic therapy in rural settings [54]. This strengthens the argument for dual dispatch and would potentially allow the MSU to attend more patients. The development of this MSU pathway has focused on ischaemic strokes, however we recognise the potential benefits of MSUs for haemorrhagic strokes, with some limited evidence around potential treatments including acute blood pressure management and the reversal of anticoagulation [55]. However, the European Stroke Association currently advise that further randomised control trials are required in relation to blood pressure lowering [56] and a recent study implementing blood pressure lowering and reversal of anticoagulation on an MSU had no statistical significant outcome differences [57]. Further, other developments regarding haemorrhagic stroke treatment, with current trials exploring a medication used to control bleeding [58], would increase the potential types of treatment available by the MSU, which may impact its dynamic location and overall cost-effectiveness. Therefore, as this study was predominantly interested in how an MSU pathway would work within the English and Welsh NHS, and the uncertainty regarding haemorrhagic stroke treatment, particularly within an MSU, this was not explored in detail but recognised as a potential development that may influence future implementation of MSUs. Other advancements, such as in artificial intelligence for reading scans [59] could also improve the efficiency and improve the time to diagnosis and treatment decisions, and improved telemedicine, particularly resolving connectivity concerns in rural areas, may also influence future implementation. In the UK the East of England Ambulance Service is currently trialling new technology, which incorporates the use of 4G, 5G and satellite connections [60]. Lastly, developments in the use of biomarkers for identification of LVOs offers another potential advancement which could be implemented on an MSU, allowing for quicker diagnosis and transportation to the CSC for treatment [61].

### Limitations

The study aimed to recruit commissioners of healthcare services within the English and Welsh NHS to the study but was unsuccessful due to uncertainty regarding how MSUs could be commissioned. Further, the study was limited to only one participant from Wales, despite significant efforts by the research team to increase this representation. Whilst a limitation of the study, it is also informative in itself and highlights the need for additional work with commissioners around future commissioning and implementation of MSUs should they be deemed potentially cost-effective. An underlying assumption of this study was that MSUs would be commissioned by the NHS however it is possible that other funding models, such as charitable or industry funding, could be an option which may require further consideration.

## Conclusion

The co-designed MSU pathway provides a foundation for MSU implementation at scale in the English and Welsh NHS. Recognising the complexity of implementing innovations in healthcare, the MSU pathway has been co-designed so that it can be subjected to necessary local and regional modifications. These modifications may address identified uncertainties in the pathway and mitigate trade-offs made during its development. Future developments in acute stroke care may help to mitigate these challenges.

## Electronic supplementary material

Below is the link to the electronic supplementary material.

Supplementary Material 1

## Data Availability

The data generated during this study are not publicly available as participants did not give consent for data sharing and the full data set contains identifying information. The authors would be happy to interrogate the data on behalf of others upon reasonable request and subject to necessary ethical approvals.

## Abbreviations

MSU: Mobile Stroke Unit
NHS: National Health Service
NGT: Nominal Group Technique
UK: United Kingdom
CT: Computed Tomography
CT-A: Computed Tomography Aangiography
IVT: Intravenous Thrombolysis
MT: Mechanical Thrombectomy
CSC: Comprehensive Stroke Centre
PPI: Patient and Public Involvement
LVO: Large Vessel Occlusion
HEMS: Helicopter Emergency Medical Services
