# Supplementary material for: Co-design of a Mobile Stroke Unit pathway highlights uncertainties and trade-offs for viable system-wide implementation in the English and Welsh NHS
Source: BMC Emerg Med. 2025 Jun 8;25:97. doi: 10.1186/s12873-025-01243-7 (PMC12147245; doi:10.1186/s12873-025-01243-7)
Supplement: Supplementary file 1 — Supplementary Material 1 [file 12873_2025_1243_MOESM1_ESM.docx]

# Workshop plan

**Workshop 1: Pathway development**

## Introduction: Presentation to participants (15 minutes)

- The purpose of the research
- Introduction of the research method – Focus Group with pathway mapping
- Plan and timings of the research activities
- Participant questions

**Research activity**

Research question 1

- What are the likely pathway(s) for mobile stroke units in ambulance services?

***Potential discussion points:***

- Dispatch protocols,
- Staffing/crewing,
- Role of telemedicine
- Nature of imaging,
- Treatment eligibility criteria,
- Protocols,
- Infrastructure costs,
- Alternative or interacting care pathways

Taking the above discussion into consideration, discuss and map what a pathway might look like

Does anything discussed introduce or address potential inequalities in access?

Research question 2

- What parameters should be considered when modelling mobile stroke units?

*Based on the previous discussion plus consideration of the developed pathway(s), particularly where similarities exist.*

*Present initial model parameters (not results of model) that have been determined by the study team.*

**Potential discussion points:**

- Process outcomes/time on scene
- Clinical outcomes (eg modified ranking scale)
- Patient reported outcomes
- Costs (as per infrastructure costs above)
- Number of dispatches / patients seen / number of strokes (valid) / numbers treated (including different treatment types)
- Destinations
- Comparators (local ambulance / stroke service performance). Any other comparators?
- Adverse events? (eg thrombolysis) and how this would affect patient outside hospital. Bleed, reaction
- Discussion to consider the developed pathway(s) discussion
- Participants may provide queries / express uncertainty, which become tasks for the research team to explore outside workshops, to bring back to future workshops

Discussion to consider the developed pathway(s) discussion

Participants may provide queries / express uncertainty, which become tasks for the research team to explore outside workshops, to bring back to future workshops

**Workshop 2: Nominal Group Technique stage 1**

## Introduction

- Recap purpose of the research and previous workshop
- Plan and timings of the research activities
- Participant questions

Introduction: Presentation to participants

- Summary of outcome from previous workshop including overview of initial pathway
- Overview of modelling results to date
- Overview of health economics result to date

Research question 1, building on previous outcomes and modelling to date

- What are the likely pathway(s) for mobile stroke units in ambulance services?

*Note: emphasis will be on amending the previously designed pathway, incorporating the provisional results of the modelling.*

Research question 2

- What parameters should be considered when modelling mobile stroke units?

*Open discussion about quantitative modelling to date, feedback on the results and discussions on what should be included.*

**Potential discussion points:**

- Are the parameters sufficient – should anything else be included or anything excluded?
- Value ranges (timings) of the parameters
- Understandability of results

Research question 3

- What should be included for the health economics of mobile stroke units?

*Open discussion about health economics, assumptions used to date and how to present health economics data.*

**Potential discussion points:**

- Assumptions made on health economics data – should anything else be included or anything excluded?
- Measuring and conveying health economic data
- Wider costs and savings associated with mobile stroke units
- What should mobile stroke unit costs/savings be compared against

**Workshop 3: Nominal Group Technique stage 2**

## Introduction

- Recap purpose of the research
- Recap research method - Nominal Group Technique (NGT)
- Plan and timings of the research activities
- Participant questions

Introduction: Presentation to participants

- Summary of outcome from previous workshop
- Overview of modelling results to date
- Overview of health economics results to date.

Research question 1, building on previous outcomes and modelling to date

- What are the likely pathway(s) for mobile stroke units in ambulance services?

Completion of survey on individual mobile stroke unit pathway elements followed by discussion of non-consensus items (set at ≥80%).

Research question 2

- What parameters should be considered when modelling mobile stroke units?

Completion of survey on value (timings) of parameter settings in the quantitative modelling followed by discussion of non-consensus items (set at ≥80%).

Research question 3

- What should be included for the health economics of mobile stroke units?

Completion of survey on what inequalities and population comparisons health economics should focus on followed by discussion of non-consensus items (set at ≥80%).
